# P29. T-cell responses to oncogenic Merkel cell polyomavirus proteins distinguish Merkel cell carcinoma patients from healthy donors

**DOI:** 10.1186/2051-1426-2-S2-P20

**Published:** 2014-03-12

**Authors:** L Lyngaa, NW Pedersen, D Schrama, CA Thrue, D Ibrani, O Met, P thor Straten, P Nghiem, JC Becker, SR Hadrup

**Affiliations:** 1 Herlev University Hospital, Center for Cancer Immune Therapy, Herlev, Denmark; 2 Medical University of Graz, General Dermatology, Graz, Austria; 3 University of Washington, Department of Medicine/Dermatology, Seattle, WA, USA

## Purpose

Merkel cell carcinoma (MCC) is a highly aggressive skin cancer with strong evidence for viral carcinogenesis. The association of MCC with the Merkel cell polyomavirus (MCPyV) may explain the explicit immunogenicity of MCC. Indeed, MCPyV-encoded proteins are likely targets for cytotoxic immune responses to MCC as they are both foreign to the host and necessary to maintain the oncogenic phenotype. However, to date only a single MCPyV-derived CD8 T-cell epitope have been described, thus impeding specific monitoring of T-cell responses to MCC.

## Method

To overcome this limitation, we scanned the MCPyV oncoproteins large T and small T antigen and the virus-capsid protein VP1 for potential T-cell epitopes, and tested for major histocompatibility complex (MHC) class I affinity. We confirmed the relevance of these epitopes using a high-throughput platform for T-cell enrichment and combinatorial encoding of MHC class I multimers.

## Results

In peripheral blood from 38 MCC patients and 30 healthy donors we identified 53 MCPyV-directed CD8+ T-cell responses against 35 different peptide sequences. Strikingly, T-cell responses against oncoproteins were exclusively present in MCC patients, but not in healthy donors. We further demonstrate both the processing and presentation of the oncoprotein-derived epitopes, as well as the lytic activity of oncoprotein-specific T cells towards MHC-matched MCC cells. Demonstrating the presence of oncoprotein-specific T cells among tumour infiltrating lymphocytes *ex vivo* further substantiated the relevance of the identified epitopes.

## Conclusion

These T-cell epitopes represent ideal targets for antigen specific immune therapy of MCC, and enables tracking and characterisation of MCPyV specific immune responses.

